# Reference values of 25-hydroxyvitamin D revisited: a position statement from the Brazilian Society of Endocrinology and Metabolism (SBEM) and the Brazilian Society of Clinical Pathology/Laboratory Medicine (SBPC/ML)

**DOI:** 10.20945/2359-3997000000258

**Published:** 2020-05-27

**Authors:** Carolina Aguiar Moreira, Carlos Eduardo dos S Ferreira, Miguel Madeira, Barbara Campolina Carvalho Silva, Sergio Setsuo Maeda, Marcelo Cidade Batista, Francisco Bandeira, Victória Z. Cochenski Borba, Marise Lazaretti-Castro

**Affiliations:** 1 Departamento de Metabolismo Ósseo Sociedade Brasileira de Endocrinologia e Metabologia Brasil Departamento de Metabolismo Ósseo, Sociedade Brasileira de Endocrinologia e Metabologia (SBEM), Brasil; 2 Hospital de Clínicas Universidade Federal do Paraná Curitiba PR Brasil Serviço de Endocrinologia e Metabologia do Hospital de Clínicas da Universidade Federal do Paraná (SEMPR), Curitiba, PR, Brasil; 3 Laboratório PRO Unidade de Histomorfometria Óssea Fundação Pró-Renal Curitiba PR Brasil Laboratório PRO, Unidade de Histomorfometria Óssea, Fundação Pró-Renal, Curitiba, PR, Brasil; 4 Sociedade Brasileira de Patologia Clínica/Medicina Laboratorial Brasil Sociedade Brasileira de Patologia Clínica/Medicina Laboratorial (SBPC/ML); 5 Departamento de Patologia Clínica Hospital Israelita Albert Einstein São Paulo SP Brasil Medicina Diagnóstica e Ambulatorial (MDA), Departamento de Patologia Clínica, Hospital Israelita Albert Einstein, São Paulo, SP, Brasil; 6 Laboratório Central Escola Paulista de Medicina Universidade Federal de São Paulo São Paulo SP Brasil Disciplina de Medicina Laboratorial, Laboratório Central, Escola Paulista de Medicina, Universidade Federal de São Paulo (EPM-Unifesp), São Paulo, SP, Brasil; 7 Universidade Federal do Rio de Janeiro Rio de Janeiro RJ Brasil Unidade de Endocrinologia, Universidade Federal do Rio de Janeiro (UFRJ), Rio de Janeiro, RJ, Brasil; 8 Unidade de Endocrinologia Hospital Felício Rocho e Santa Casa de Belo Horizonte Belo Horizonte MG Brasil Unidade de Endocrinologia, Hospital Felício Rocho e Santa Casa de Belo Horizonte, Belo Horizonte, MG, Brasil; 9 Centro Universitário de Belo Horizonte Belo Horizonte MG Brasil Disciplina de Endocrinologia, Centro Universitário de Belo Horizonte (UNI-BH), Belo Horizonte, MG, Brasil; 10 Escola Paulista de Medicina Universidade Federal de São Paulo São Paulo SP Brasil Disciplina de Endocrinologia, Escola Paulista de Medicina, Universidade Federal de São Paulo (EPM-Unifesp), São Paulo, SP, Brasil; 11 Divisão de Endocrinologia e Diabetes Faculdade de Medicina Universidade de Pernambuco Recife PE Brasil Divisão de Endocrinologia e Diabetes, Faculdade de Medicina, Universidade de Pernambuco (UPE), Recife, PE, Brasil

**Keywords:** Vitamin D, 25-hydroxyvitamin D, reference range, vitamin D intoxication

## Abstract

Hypovitaminosis D is a common condition with a negative impact on health. This statement, prepared by experts from the Brazilian Society of Endocrinology and Metabolism and the Brazilian Society of Clinical Pathology/Laboratory Medicine, includes methodological aspects and limitations of the measurement of 25-hydroxyvitamin D [25(OH)D] for identification of vitamin D status, and identifies individuals at increased risk for deficiency of this vitamin in whom 25(OH)D measurement is recommended. For the general population, 25(OH)D levels between 20 and 60 ng/mL are considered normal, while individuals with levels below 20 ng/mL are considered to be vitamin D deficient. This statement identifies potential benefits of maintaining 25(OH)D levels > 30 ng/mL in specific conditions, including patients aged > 65 years or pregnant, those with recurrent falls, fragility fractures, osteoporosis, secondary hyperparathyroidism, chronic kidney disease, or cancer, and individuals using drugs with the potential to affect the vitamin D metabolism. This statement also calls attention to the risk of vitamin D intoxication, a life-threatening condition that occurs at 25(OH)D levels above 100 ng/mL

## INTRODUCTION

Hypovitaminosis D is highly prevalent according to international and Brazilian studies, independent of the region evaluated ( 1 , 2 ). However, the prevalence rates of this condition vary according to the reference values established for 25-hydroxyvitamin D [25(OH)D], the metabolite measured to determine the vitamin D status ( 3 ).

In 2010, the US Institute of Medicine proposed an increase in the daily recommended amount of vitamin D for healthy adults from 200 IU to 600 IU. The Institute also considered 25(OH)D concentrations < 20 ng/mL to be potentially harmful for the general American population ( 4 ). Shortly after that, the Endocrine Society established the value of 30 ng/mL (instead of 20 ng/mL) as the lower limit of normal for 25(OH)D levels ( 5 ). Based on publications by these two institutions, along with review of national and international literature, the Brazilian Society of Endocrinology and Metabolism ( *Sociedade Brasileira de Endocrinologia e Metabologia* – SBEM) established in 2014 the 25(OH)D concentration of ≥ 30 ng/mL as desirable for populations at risk of harmful consequences from hypovitaminosis D ( 3 ). Since then, many studies on the effects of vitamin D supplementation have been published. At the same time, an intense debate has developed around the establishment of reference values for 25(OH)D, and new guidelines have emerged proposing levels between 20-30 ng/mL ( 6 - 9 ). This discussion prompted a review of the topic in 2017 by SBEM along with the Brazilian Society of Clinical Pathology/Laboratory Medicine (SBPC/ML) ( 10 ). Considering new evidence that has emerged since the review, the aim of this article is to present a critical evaluation of the current methodology for 25(OH)D measurement, report the groups most susceptible to the deficiency, and identify clinical situations in which low vitamin D concentrations are harmful and, thus, 25(OH)D levels above 30 ng/mL are recommended. A secondary objective of this article is to discuss the upper limit values of 25(OH)D that are deemed safe and the risks and causes of vitamin D intoxication.

Importantly, this document is intended to be a guide for clinicians dealing with specific populations and was written by a task force comprising experts from both societies who, by interpreting the scientific evidence in light of their broad research and clinical experience, contributed with their undeniable expert opinions. These concepts may change as new evidence emerges.

## METHODOLOGICAL ASPECTS OF 25(OH)D MEASUREMENT

Vitamin D comprises a group of fat-soluble secosteroids. In humans, vitamin D_3_ (cholecalciferol) is produced mainly in the skin from exposure of 7-dehydrocholesterol to type B ultraviolet radiation (wavelength 290 to 315 mn) from sunlight. In plants and fungi, vitamin D_2_ (ergocalciferol) is synthesized by the action of ultraviolet radiation on ergosterol. Both vitamins D_3_ and D_2_ are obtained from diet, mainly from the consumption of fatty fish, cod liver oil, egg yolk, wild mushrooms, and fortified products (milk, cereals, etc.), although compared with cutaneous production, the diet is a much less important source of vitamin D for the body ( 11 , 12 ).

Vitamin D_3_ or D_2_ is initially metabolized in the liver and converted to 25(OH)D by 25-hydroxylase (CYP2R1) enzyme activity. Subsequently, 25(OH)D undergoes a second hydroxylation in the kidneys, mediated by the enzymes 1α-hydroxylase (CYP27B1) and 24,25-hydroxylase (CYP24A1), producing the metabolites 1,25-dihydroxyvitamin D [1,25(OH)_2_D] and 24,25-dihydroxyvitamin D [24,25(OH)_2_D], respectively. 1,25(OH)_2_D, the active metabolite of vitamin D, can promote bone reabsorption, stimulate intestinal absorption of calcium and phosphorus and inhibit urinary excretion of these ions. 24,25(OH)_2_D is the main product of 25(OH)D catabolism, and its concentration correlates strongly with the concentration of 25(OH)D. In the bloodstream, 25(OH)D, 1,25(OH)_2_D, and 24,25(OH)_2_D metabolites circulate mainly (85%-90%) bound to vitamin D-binding protein (DBP) and, to a lesser extent (10%-15%), to albumin and lipoproteins. Only a small fraction (≤1%) of 25(OH)D circulates in a free form. The free fraction plus the albumin-bound fraction are collectively named “bioavailable fraction”, since 25(OH)D is able to easily dissociate from these albumin proteins due to low affinity, becoming available to act on target cells. In most cells, free 25(OH)D is believed to cross cell membranes by simple diffusion and without mediation by carrying proteins. In renal tubular cells, 25(OH)D linked to DBP can be internalized by endocytosis mediated by the megalin/cubulin complex present in cell membranes. Once in the cytoplasm, free 25(OH)D [or 25(OH)D dissociated from DBP, if internalized while linked to this globulin] is converted to 1,25(OH)_2_D to further interact with intranuclear receptors ( 11 , 12 ).

Production of 1,25(OH)_2_D is regulated by several direct and indirect mechanisms. 1,25(OH)_2_D directly inhibits the activity of 1α-hydroxylase (CYP27B1), decreasing further production of 1,25(OH)_2_D. Additionally, 1,25(OH)_2_D suppresses the secretion of PTH by the parathyroid glands; since PTH is an inducer of 1α-hydroxylase (CYP27B1), this mechanism inhibits the activity of this enzyme indirectly. Increased levels of 1,25(OH)_2_D also stimulate renal production of fibroblast growth factor 23 (FGF-23; a phosphaturic factor), which in turn inhibits the activity of 1α-hydroxylase (CYP27B1). Finally, some studies suggest that the dietary intake of calcium and phosphorus can also suppress the expression of this enzyme ( 12 ).

25(OH)D is the main vitamin D metabolite, and its measurement is considered the best indicator of the vitamin D reserve in the body. Due to its relatively long half-life (2-3 weeks), the circulating levels of 25(OH)D show little fluctuation, reflecting the combination of dietary intake and cutaneous vitamin D production ( 12 , 13 ).

The main techniques for measurement of 25(OH)D are ligand assays and chromatographic methods associated with ultraviolet detection or tandem mass spectrometry ( 12 - 14 ). Most laboratories use binding assays since these assays involve methods that are generally automated, inexpensive, fast, and easy to perform ( 15 ). These assays include a first step in which 25(OH)D is dissociated from its carrier proteins. In a second step, the 25(OH)D in the sample competes with an analogue for the same sites of the ligand’s assay [anti-25(OH)D antibodies and DBP]. Either the analogue or the ligand is conjugated to a tracer (usually a chemiluminescent or electrochemiluminescent tracer) ( 12 ). Despite their practicality, these assays have some limitations, including different specificity of the assay’s ligand for 25(OH)D_2_ and 25(OH)D_3_ and cross-reactivity with vitamin D metabolites [mainly 24,25(OH)_2_D] ( 16 , 17 ). Additionally, for accurate measurement of its total concentration, 25(OH)D must be completely dissociated from its binding proteins prior to the analysis, which may not occur in some situations, particularly in individuals with increased DBP (women who are pregnant or using estrogens) ( 18 ). All these factors represent potential sources of error in 25(OH)D measurement.

Liquid chromatography, coupled with tandem mass spectrometry (LC-MS/MS), is considered the gold standard for 25(OH)D measurement due to its high precision and specificity and low analytical interference ( 12 - 14 ). Two methods developed either by the National Institute of Standards and Technology (NIST, USA) or by Ghent University (Belgium) are considered as references by the Joint Commission for Traceability in Laboratory Medicine ( 16 , 19 ). Limitations to the widespread use of LC-MS/MS in laboratories include the high cost of acquiring and maintaining the equipment, the need for specialized professionals to develop and validate the method, and less automation, requiring more labor and time for each measurement. Several LC-MS/MS assays are also prone to C3-epimer interference, which could result in falsely increased 25(OH)D levels. This occurs mainly in children under the age of 1 year, in whom C3-epimer levels are higher ( 12 - 14 , 16 ).

Despite advances in technological developments and methodological standardizations in recent years, there are still considerable variations in 25(OH)D levels obtained in different trials, which may impact the clinical interpretation of the results ( 20 ). In laboratory practice, up to 20% of variation may occur between different methods due to several factors: analytical inaccuracy and imprecision; matrix effect mainly caused by lipemia and variable DBP levels in the sample; variable and incomplete DBP-25(OH)D dissociation, especially in samples with high DBP levels; different reactivity of the assay ligand for 25(OH)D_2_ and 25(OH)D_3_; and cross-reactions, mainly with the C3-epimer and 24,25(OH)_2_D ( 20 - 24 ).

The main challenge for the diagnostic market is to achieve better standardization across ongoing trials, which would provide a better comparison of results obtained in different laboratories and clinical studies. This would allow us to determine with greater certainty which individuals actually have vitamin D deficiency and to establish toxic levels with negative health impacts. Some programs, such as the Vitamin D Standardization Program (USA) and the Vitamin D External Quality Assessment Scheme (DEQAS, UK), directly target this standardization in an attempt to reduce the differences between methods ( 15 , 19 ). From a methodological standpoint and considering the current analytical variation between different methods, researchers like Binkley and Carter – both responsible for the most important proficiency testing survey currently available (DEQAS) and for the publication of several studies comparing different 25(OH)D assays – have suggested that 25(OH)D levels should be maintained between 30-40 ng/mL to ensure concentrations greater than 20-30 ng/mL, since no toxic effects occur at these levels and the real 25(OH)D concentration in the samples is generally unknown ( 19 ).

Some studies have recently suggested that measurements of the free or bioavailable 25(OH)D fraction correlate better with bone parameters than total 25(OH)D measurements, especially in some subgroups like postmenopausal women and patients with osteoporosis, chronic kidney disease on dialysis, or cirrhosis ( 25 , 26 ). These fractions can be estimated using Vermeulen’s formula from the values of total 25(OH)D, DBP, and albumin and their affinity constants. However, this formula is not widely accepted because it has not been validated against a reference method and is subject to the limitations of the total 25(OH)D and DBP assays used in the calculation. Depending on the type of antibody used (monoclonal versus polyclonal), DBP immunoassays may not recognize all circulating DBP isoforms, resulting in lower values of this protein and overestimating the free and bioavailable fractions. In studies comparing DBP levels in African Americans and Caucasian Americans, DBP measured by monoclonal immunoassay was lower in African Americans, whereas in other studies in which DBP was measured by polyclonal immunoassay or LC-MS/MS, this difference was not found ( 27 , 28 ). Other methods that allow direct measurement of the free 25(OH)D fraction include equilibrium dialysis, ultrafiltration, and some commercial immunoassays; however, none of these methods has been widely validated. Thus, these measurements are rarely available in clinical laboratories in general and, at present, have very limited indications in clinical practice.

Measurement of 1,25(OH)_2_D, the active metabolite of vitamin D, is generally not recommended in the assessment of the nutritional status of vitamin D due to its short half-life (4-6 hours) and a rigid control of its serum levels by calcium, phosphorus, PTH, and FGF-23 ( 12 , 13 ). Normal or even elevated levels of 1,25(OH)_2_D are often found in individuals with vitamin D deficiency due to associated secondary hyperparathyroidism, with consequent increased expression of the enzyme 1α-hydroxylase (CYP27B1) and increased production of 1,25(OH)_2_D. In contrast, the activity of the enzyme 25-hydroxylase (CYP2R1) is fundamentally dependent on the availability of its substrate (vitamin D) and is not influenced by its product [25(OH)D]. Because of this, 25(OH)D is a more reliable indicator of vitamin D stored in the body. In addition, since the circulating levels of 1,25(OH)_2_D are 1,000 times lower than those of 25(OH)D, measurement of 1,25(OH)_2_D is much more complex, and no method or reference material is currently available for that. Assays used for such measurement include radioimmunoassay with sample extraction and/or chromatography, some recently implemented automated immunoassays, and LC-MS/MS ( 12 , 13 ). Measurement of 1,25(OH)_2_D is only useful in some specific situations, including chronic renal failure, oncogenic osteomalacia, hereditary forms of rickets (hypophosphatemic, vitamin D resistant or associated with 1α-hydroxylase deficiency), and granulomatous diseases (sarcoidosis and some types of lymphoma).

## CLINICAL CONDITIONS AT INCREASED RISK FOR VITAMIN D DEFICIENCY

The metabolite 25(OH)D is not the active form of vitamin D but is universally accepted as the main marker of vitamin D status ( 3 ). Due to growing availability of information about the consequences of vitamin D deficiency and high rates of this condition, there has been an increasing number of requests for the assessment of vitamin D status, many of which are questionable.

Plasma 25(OH)D measurement is recommended in groups with conditions at risk for vitamin D deficiency, listed in Table 1 . These clinical conditions can be grouped according to the pathophysiology of the vitamin deficiency as (A) reduced production by insufficient skin synthesis or inadequate hepatic and renal transformation, (B) increased degradation or consumption, (C) malabsorption and/or intestinal loss.

**Table 1:** Main clinical conditions associated with vitamin D deficiency

Insufficient production: cutaneous, hepatic, or renal	Increased metabolization/consumption	Reduced intestinal absorption
Older age	Medications: anticonvulsant agents (phenobarbital, carbamazepine, diphenylhydantoin), ketoconazole isoniazid antiretrovirals (efavirenz, tenofovir) antibiotics	Intestinal malabsorption: inflammatory diseases, celiac disease, Crohn’s disease, cystic fibrosis, pancreatic insufficiency
Dark skin	Inflammatory conditions (SLE, RA, tuberculosis)	Bariatric surgery, pancreatic or intestinal resections
Physical barriers (sunscreen, clothing, glass)	Primary hyperparathyroidism	Medications: orlistat, cholestyramine
Obesity	Osteoporosis treatment with teriparatide or PTH (1-84)	
Reduced solar exposure (pregnancy, risk of skin cancer, post-transplantation, SLE)		
Reduced 25(OH)D production: severe hepatic impairment		
Reduced 1,25(OH)_2_D production/action: chronic kidney disease and vitamin D-dependent rickets type I and II, X-linked hypophosphatemic rickets, and other conditions associated with excessive FGF-23		

SLE: systemic lupus erythematosus; RA: rheumatoid arthritis; 25(OH)D: 25-hydroxyvitamin D; 1,25(OH)_2_D: 1,25 dihydroxyvitamin D; FGF-23: fibroblast growth factor 23.

- **(A) Insufficient production** of vitamin D occurs in the elderly (skin aging) ( 29 , 30 ), in individuals with dark skin, due to physical barriers (religious clothing, sunscreen, glass), in individuals who are bedridden or restricted to closed environments (neurological, psychiatric, or institutionalized patients), in obesity (mixed causes), and in pregnancy ( 31 - 34 ). The occurrence of 25-hydroxylation of vitamin D in the liver may be compromised in states of severe hepatic impairment. In renal insufficiency, vitamin D-dependent rickets type I, and conditions with excessive FGF-23, the production of 1,25(OH)_2_D is reduced due to impaired 1-alpha-hydroxylase activity, in which, low 25(OH)D concentrations may further compromise bone metabolism ( 35 ).
- **(B) Increased degradation** of vitamin D and its metabolites may be caused by medications that activate hepatic lysosomal enzymes, like anticonvulsant agents (carbamazepine, phenobarbital, hydantoin), antiretrovirals (efavirenz, tenofovir), antibiotics, and antifungal agents (isoniazid, ketoconazole) ( 36 , 37 ). Increased degradation of the vitamin may also occur due to increased consumption of 1,25(OH)_2_D by inflammatory cells [as in rheumatoid arthritis (RA), systemic lupus erythematosus (SLE), and tuberculosis]. Furthermore, low 25(OH)D may occur from increased 25(OH)D to 1,25(OH)_2_D conversion by increased PTH in primary hyperparathyroidism and during treatment with PTH (teriparatide) ( 38 - 42 ); this can occur due to increased activity of renal 1-alpha-hydroxylase ( 43 ), the enzyme responsible for this conversion. On the other hand, increased 1,25(OH)_2_D can induce CYP24A1 activity, which converts 25(OH)D into its inactive form, 24,25(OH)_2_D. Still, other factors may also contribute to low 25(OH)D levels in hyperparathyroidism ( 44 ).
- **(C) Intestinal malabsorption** causes vitamin D to be eliminated in the feces along with fat, since vitamin D is part of the enterohepatic cycle. This occurs in disorders with intestinal inflammation or malabsorption, like celiac disease, cystic fibrosis, Crohn’s disease, and pancreatic insufficiency. It may also occur with medications that limit the absorption of vitamin D, such as cholestyramine and orlistat, and conditions with iatrogenic malabsorption following bariatric surgery and pancreatic or intestinal resections ( 1 , 45 ).

## WHICH CONDITIONS COULD BENEFIT FROM 25(OH)D CONCENTRATIONS ABOVE 30 NG/ML?

According to the Institute of Medicine, 25(OH)D concentrations below 20 ng/mL in the general population are considered low ( 4 ). In contrast, evidence suggests that 25(OH)D concentrations maintained above 30 ng/mL in some clinical situations are beneficial to the patient, especially in reducing the risk of fractures. The main clinical conditions benefiting from 25(OH)D levels > 30 ng/mL are described below ( Table 2 ).

**Table 2:** Clinical conditions and groups that benefit from 25-hydroxyvitamin (25[OH]D) concentrations above 30 ng/mL

Groups	Clinical Conditions
Elderly (> 65 years) Pregnant women	Osteoporosis (primary or secondary) Fractures due to fragility
	Metabolic bone diseases (osteomalacia, osteogenesis imperfecta, primary hyperparathyroidism) Secondary hyperparathyroidism
	Sarcopenia Recurring falls Chronic renal disease
	Malabsorption syndrome
	Liver failure
	Diabetes mellitus type 1
	Cancer

### Elderly and falls

Due to lifestyle habits, polypharmacy, multiple comorbidities, and reduced efficacy of skin production of vitamin D, elderly individuals comprise one of the most important groups at risk of vitamin D deficiency and consequent secondary hyperparathyroidism ( 29 - 31 ). Low 25(OH)D concentrations are associated with increased risk of fractures and falls, especially in frail and institutionalized elderly ( 46 ). Since secondary hyperparathyroidism is very frequent in the elderly population and has harmful consequences (especially for bone mass), 25(OH)D concentrations above 30 ng/mL have been recommended for normalization of PTH levels in this population ( 32 ). This finding was similar to results from Brazilian studies, which found a threshold of around 30-32 ng/mL for 25(OH)D; levels lower than these were associated with increased serum PTH levels ( 47 , 48 ). However, a study evaluating 488 elderly Caucasian women was unable to find a correlation between 25(OH)D and PTH and, therefore, to define a threshold value for 25(OH)D in this population ( 49 ).

A pooled analysis concluded that vitamin D supplementation at doses ≥ 800 IU/day was associated with a reduced risk of vertebral and femoral fractures in individuals aged ≥ 65 years ( 50 ). The authors also observed that patients older than 85 years and those with lower 25(OH)D levels were the ones benefiting most from vitamin D supplementation. Another recent meta-analysis ( 51 ) was unable to confirm these findings but received negative criticism regarding its methodology, including the selection of the studies and lack of adjustments in terms of the evaluation of adherence to the interventions ( 52 ).

Elderly individuals have an increased prevalence of sarcopenia and, thus, a higher risk of falls and fractures. Presence of the vitamin D receptor (VDR) has been demonstrated in skeletal muscle precursor cells, while the number of VDRs in muscle appears to decline with aging ( 53 ). A study evaluating muscle fibers after treatment with vitamin D_3_ 4,000 IU in 21 elderly women with a baseline level of 25(OH)D of 18 ng/mL demonstrated increased intramyonuclear VDRs in type II muscle fibers compared with a placebo group. In addition, a 30% increase in the cross-sectional area was observed in muscle fibers along with intramyonuclear VDR concentration after treatment with vitamin D_3_ ( 54 , 55 ). A similar finding occurs with aging and suggests that elderly individuals with low vitamin D levels may have exacerbated muscle atrophy ( 56 ). A multicenter Italian study evaluating 401 elderly women (mean age 66.9 years) demonstrated that those with vitamin D deficiency had a significant reduction in appendicular muscle strength and physical performance compared with women with 25(OH)D levels above 30 ng/mL ( 57 ), reinforcing the occurrence of a deleterious effect of vitamin D deficiency on the muscle ( 58 ).

A Brazilian study has shown significant increases of 16.4% and 24.6% in the strength of hip flexors and knee extensors, respectively, after 6 months of cholecalciferol supplementation in elderly patients without any regular physical activity ( 59 ).

Regarding vitamin D supplementation, a systematic review by Beaudart and cols. concluded that it was associated with a significant increase in overall muscle strength, more evident in individuals > 65 years and in those with very low (< 12 ng/mL) initial 25(OH)D values ( 60 ). Similarly, a recent randomized clinical trial demonstrated that administration of vitamin D for 6 months, which resulted in a mean 25(OH)D level of 47 ng/mL, had a positive effect on increasing muscle mass and physical strength, an effect that was independent of physical activity ( 61 ).

These studies emphasize the fact that elderly individuals have, in addition to a higher risk of hypovitaminosis D, important clinical consequences, such as increased risk of falls and bone fragility, which increase the risk of fractures. According to a Cochrane review, vitamin D supplementation reduces the risk of falls in institutionalized individuals – mostly in vitamin D deficient ones – but has little effect on the risk of falls among outpatients ( 62 ). In contrast, a study with high doses of vitamin D, which resulted in increased 25(OH)D levels, showed the opposite effect, *i.e.* , an increased risk of falls in elderly individuals ( 63 ). These results confirm that the administration of vitamin D at higher doses (as “bolus doses”) has no skeletal benefits, whereas daily or weekly doses are more physiological and are thus recommended.

Recently, the Vitamin D Assessment (ViDA) study, with more than 5,000 adults, demonstrated no effect of a high monthly vitamin D dose on falls ( 64 ). Most participants had adequate 25(OH)D values prior to the intervention, which may have influenced the results.

A Brazilian study by Cangussu and cols. demonstrated an effect of vitamin D supplementation, in which an increase of 25(OH)D levels to 27.5 ± 10.4 ng/mL was associated with a reduction in the number of falls and improvement of postural balance in a group of postmenopausal women compared with a placebo group with 25(OH)D levels of 13.8 ± 6.0 ng/mL ( 65 ). In this same group of patients, the authors had previously demonstrated a positive effect of vitamin D supplementation [and therefore increased serum 25(OH)D levels] on increasing lower limb muscle strength by 25.3% compared with the placebo group, which presented a considerable loss in this parameter, suggesting a role of vitamin D in preventing sarcopenia ( 66 ). In a recent review, Bouillon and cols. concluded that daily supplementation with modest doses of vitamin D in elderly subjects with vitamin D deficiency may modestly improve muscle function and balance and decrease the risk of falls ( 67 ).

In summary, vitamin D supplementation can have beneficial effects, deleterious effects, or no effect at all on the risk of falls, depending on the baseline 25(OH)D levels and the dose of vitamin D.

### Pregnancy

Vitamin D deficiency is highly prevalent during pregnancy ( 68 , 69 ) and occurs more frequently in the first trimester. Serum 25(OH)D levels measured at the end of pregnancy, compared with levels measured early in pregnancy, correlate better with clinical outcomes, especially with increased risk of preterm delivery ( 70 ). Vitamin D supplementation leading to serum 25(OH)D concentrations ≥ 30 ng/mL has demonstrated positive effects on genes related to preeclampsia ( 71 , 72 ). In a randomized controlled trial, correction of low vitamin D levels to mean concentrations of approximately 30 ng/mL significantly reduced the risk of preeclampsia and intrauterine growth retardation ( 73 ). A similar finding was demonstrated in a recent Brazilian study ( 74 ). In contrast, other studies have not demonstrated benefits from vitamin D supplementation on the risk of preeclampsia or hypertension in pregnancy ( 71 ).

Levels of 25(OH)D have also been correlated with prematurity. The risk of prematurity has been shown to be 3.8 times higher in pregnant women with serum 25(OH)D levels below 20 ng/mL compared with those with levels above 40 ng/mL ( 70 , 75 ).

Studies analyzing the association between 25(OH)D concentrations and birth weight have suggested a negative correlation between the vitamin concentrations and low birth weight ( 70 , 72 ). A recent meta-analysis of 54 studies has shown that offspring of mothers with 25(OH)D < 55 nmol/L (22 ng/mL) are at increased risk of low birth weight and anthropometric abnormalities ( 33 ). The study also showed an increased risk of preterm birth among mothers with 25(OH)D < 30 nmol/L (12 ng/mL) and lower scores in mental and language developmental tests among offspring of vitamin D insufficient mothers. Levels of 25(OH)D values above 75 nmol/L (30 ng/mL) showed no correlation with these abnormalities ( 33 ). A randomized clinical trial with vitamin D deficient expectant mothers compared the supplementation with three doses of vitamin D versus placebo during and after delivery found no difference in anthropometric measures or morbidity between groups ( 76 ).

More recently, an update of a systematic review including 30 trials (7,033 pregnant women) concluded that the supplementation with vitamin D alone during pregnancy probably reduces the risk of preeclampsia, gestational diabetes, and low birth weight (with moderate-certainty evidence) compared with placebo or no intervention. The study also showed with low-certainty evidence that vitamin D supplementation has no effect in the risk of preterm birth compared with no intervention or placebo, and may reduce the risk of severe postpartum hemorrhage ( 77 ). Although the supplementation of vitamin D during pregnancy is still controversial, none of these studies showed major adverse effects associated with this approach.

### Osteoporosis and other bone diseases

Vitamin D plays a major role in calcium absorption and bone mineralization. Low 25(OH)D levels are associated with poorer bone quality and higher fracture risk ( 78 ). The combined effects of insufficient daily calcium intake and vitamin D deficiency lead to low bone mineral density (BMD) and increased prevalence of osteopenia and osteoporosis in Korean women ( 78 ). Despite conflicting evidence ( 79 ), some studies have found a significant increase in bone density and a decrease in hip and non-vertebral fractures with vitamin D supplementation alone or in combination with calcium ( 80 , 81 ). In fact, a meta-analysis supports the use of daily vitamin D to reduce the incidence of osteoporotic non-vertebral, non-hip fractures in elderly women ( 80 ). Vitamin D with calcium appears to achieve benefits above those attained with calcium supplementation alone for non-vertebral and non-vertebral, non-hip fractures ( 80 ). These protective effects were more pronounced in patients with low baseline 25(OH)D levels (< 10-12 ng/mL) and in a nursing home population ( 82 ). Recently, a substudy from the ViDA trial including subjects with a baseline 25(OH)D level of 12 ng/mL found that monthly doses of vitamin D_3_ of 100,000 IU for 2 years significantly attenuated the BMD loss at the femoral neck and total hip ( 83 ).

Primary hyperparathyroidism is associated with reduced BMD and greater fracture risk. Low vitamin D levels are frequent in patients with this condition ( 84 ), and evidence suggests that vitamin D deficiency in these patients is associated with more aggressive disease, greater levels of PTH and bone turnover markers, and a higher risk of hungry bone syndrome following parathyroidectomy ( 85 ). Indeed, a randomized controlled trial in patients with primary hyperparathyroidism showed that vitamin D supplementation for 6 months increased the mean serum concentration of 25(OH)D from 20 ng/mL to 38 ng/mL, which resulted in improvements in lumbar spine BMD and reductions in serum C-terminal telopeptide (CTX) concentrations, without increasing serum or urinary calcium ( 84 ). Thus, guidelines recommend serum levels of 25(OH)D to be maintained > 30 ng/mL in this group of patients ( 86 ).

Chronic hypovitaminosis D (due to insufficient vitamin D intake or sun exposure) and/or low calcium intake can induce poor bone mineralization, leading to rickets and osteomalacia. A global consensus recommends vitamin D supplementation (400 IU) to prevent nutritional rickets and osteomalacia during childhood. Serum 25(OH)D levels above 20 ng/mL seem adequate for bone mineralization in children ( 87 ).

### Secondary hyperparathyroidism

Vitamin D and PTH are two closely interrelated metabolites, and their plasma concentrations should be interpreted in combination ( 88 ). Vitamin D is an important inhibitor of PTH synthesis in the parathyroid and its deficiency is associated with elevated blood PTH concentrations, defined as secondary hyperparathyroidism. Increased PTH concentrations are associated with undesirable outcomes, especially in elderly populations, such as falls, fractures, and increased mortality ( 89 , 90 ). A prospective study in older men demonstrated that higher PTH levels were associated with an increased rate of BMD loss compared with lower PTH levels, independently of vitamin D level and renal function ( 91 ). A study evaluating femoral tomography in postmenopausal women has shown a relationship between PTH increase and cortical porosity and an increased risk of fractures ( 90 ). The relationship between vitamin D and PTH is not linear, and the threshold at which PTH begins to rise varies widely in the literature due to the use of different assays, age groups, and calcium intake in the study populations. In general, 25(OH)D concentrations are recommended to be maintained between 20 ng/mL and 40 ng/mL to prevent the development of hyperparathyroidism secondary to vitamin D deficiency. Vitamin D deficiency should be one of the first causes to be excluded in case of doubt between a diagnosis of normocalcemic primary hyperparathyroidism and secondary hyperparathyroidism. In this scenario, 25(OH)D concentrations should be maintained above 30 ng/mL prior to investigating a suspected primary normocalcemic hyperparathyroidism ( 92 ). In fact, guidelines from the Fourth International Workshop on Asymptomatic Primary Hyperparathyroidism have established a serum 25(OH)D level greater than 30 ng/mL as desirable for the diagnosis of normocalcemic primary hyperparathyroidism ( 93 ). A study has shown that an increase in 25(OH)D levels to 30 ng/mL with vitamin D supplementation in deficient women (< 20 ng/mL) is associated with a significant decrease in PTH levels, including in two participants in whom the baseline 25(OH)D levels were around 19 ng/mL (which could have been 20 ng/mL, considering the precision error of the method). These data showed a not well understood individual variation on the relationship between PTH and vitamin D, and indicated that some individuals may benefit from higher 25(OH)D levels and should not be overlooked ( 94 ).

In a study with a cohort representative of the Brazilian population, the 25(OH)D threshold for PTH elevation was below 30 ng/mL, and this correlation was most evident in elderly individuals (> 65 years) ( 95 ). Similarly, another cross-sectional study evaluating more than 300,000 paired serum PTH and 25(OH)D measurements detected a clear inverse correlation between both but found no threshold or inflection point in the curve. Levels of PTH continue to decrease as those of 25(OH)D rise and, in the study, the differences in PTH levels categorized by age range became clear. Younger individuals (< 20 years of age) have lower PTH concentrations that begin to rise when 25(OH)D concentrations are lower than 20 ng/mL. In contrast, as the prevalence of secondary hyperparathyroidism increases with age, the relationship between PTH levels and lower 25(OH)D levels becomes clearer in older individuals ( 96 ).

Other causes of secondary hyperparathyroidism that may be indirectly related to vitamin D deficiency are those leading to intestinal malabsorption such as celiac disease, cystic fibrosis, inflammatory bowel diseases, and bariatric surgery ( 97 ).

Bariatric surgery is a frequent cause of secondary hyperparathyroidism in which PTH concentrations can reach extreme levels caused by severe vitamin D deficiency combined with low intake and bioavailability of dietary and supplemental calcium. In the long term, these changes are associated with an increased risk of fractures ( 98 ). Most available literature considers the target 25(OH)D level of 30 ng/mL after bariatric surgery. Doses of vitamin D above those usually recommended may be required to adjust the 25(OH)D concentrations after bariatric surgery, which should always be accompanied by adequate calcium intake ( 98 , 99 ). Strategies for vitamin D supplementation vary broadly in the literature. Doses below 800 IU/day seem to be insufficient to reach the target 25(OH)D blood level. Many studies suggest the administration of 50,000 IU weekly plus a daily dose, but no consensus has been reached in this regard. The ideal strategy to date is to find the best treatment regimen for each patient by titrating the dose of vitamin D until optimal plasma concentrations are reached.

However, secondary hyperparathyroidism after bariatric surgery cannot be attributed to vitamin D deficiency alone. Impaired calcium absorption seems to be a very important issue hindering improvements in secondary hyperparathyroidism, even under normal 25(OH)D concentrations (> 30 ng/mL), as described by Tardio and cols. ( 34 ).

Obese individuals have lower vitamin D levels than nonobese ones, and this deficiency should be identified and corrected before bariatric surgery. The 25(OH)D levels in these patients are recommended to be maintained above 30 ng/mL before this type of surgery ( 100 , 101 ), and depending on the surgical technique, doses much higher than conventional ones may be required after surgery to meet this goal. These concentrations should be periodically evaluated, and the doses should be titrated according to blood 25(OH)D levels ( 99 ).

### Diabetes mellitus

Several studies have addressed the supplementation of vitamin D in patients with prediabetes and diabetes, showing controversies results, especially in patients with type 2 diabetes (T2DM). Recently, Pittas et al. randomized 2,423 individuals to receive vitamin D 4,000 IU or placebo, and after 2.5 years, the authors observed no reduction in the risk of T2DM with vitamin D supplementation ( 102 ). In contrast, vitamin D supplementation may have benefits on B cell function and in the immune system in type 1 diabetes, as demonstrated in a Brazilian study ( 103 ).

### Chronic renal disease

Vitamin D deficiency is prevalent among patients with chronic renal disease (CKD) treated conservatively or with dialysis, and among kidney-transplanted patients ( 104 , 105 ). A meta-analysis with more than 17,000 patients concluded that hypovitaminosis D was associated with an increased risk of all-cause mortality, especially in patients undergoing dialysis. Additionally, a 10 ng/mL increase in serum 25(OH)D levels was associated with a 21% mortality reduction, while values above 25 ng/mL were associated with lower mortality risk. Of note, no additional benefit was observed in patients with 25(OH)D values greater than 35 ng/mL ( 106 ).

The Kidney Disease Improving Global Outcomes (KDIGO) guidelines recommend measurement of 25(OH)D levels in patients with CKD and suggest that the ideal serum levels are similar to those recommended for the general population ( 105 ). Since CKD is a chronic disease associated with increased risk of fractures, 25(OH)D concentrations above 30 ng/mL would be recommended. A randomized, double-blind, placebo-controlled study evaluated the supplementation with high doses of cholecalciferol in 120 patients with stage 3-4 CKD ( 107 ). After 16 weeks, 25(OH)D serum levels were around 40 ng/mL in the treated group, which led to a significant reduction in PTH, CTX, and bone alkaline phosphatase compared with the placebo group. Even though serum 25(OH)D levels above 40 ng/mL were related to reduced bone remodeling, there was no evaluation of fracture risk. To date, no studies in this specific population have demonstrated a relationship between reduced bone remodeling and reduced risk of fragility fractures.

### Cancer

*ln vitro* studies have indicated that 1,25(OH)_2_D, the active form of vitamin D, has several antineoplastic effects, including antiproliferative and anti-inflammatory actions, inhibition of angiogenesis and metastasis, as well as stimulation of differentiation and apoptosis of malignant cells ( 108 ). Accordingly, clinical observational studies have demonstrated associations between low serum 25(OH)D concentrations at baseline and increased risk of incident malignant diseases and/or mortality from cancer ( 109 - 112 ). Specifically, 25(OH)D serum levels lower than 25 ng/mL have been associated with a greater risk of cancer death, including digestive, central nervous, pulmonary, hematological, and breast cancers ( 110 ).

In another prospective study, serum 25(OH)D concentrations greater than 38 ng/mL were associated with lower rates of incident breast cancer in women with increased risk of developing this malignancy ( 111 ). In contrast, several meta-analyses of observational studies have shown that vitamin D supplementation does not reduce the risk of incident cancers, but may decrease cancer mortality. Nevertheless, these data fail to support the hypothesis that an increase in 25(OH)D serum levels through vitamin D supplementation could reduce the incidence of cancer or improve cancer outcomes. Evidence from randomized controlled trials (RCTs) is needed to support results from observational studies. To this end, several RCTs have examined the effect of calcium and vitamin D supplementation on cancer incidence and mortality. The results, described below, are still controversial. More recently, a secondary analysis of data from the Women’s Health Initiative Calcium/Vitamin D trial showed a protective effect of calcium plus a daily dose of 400 IU of vitamin D (CaD) supplementation on the risk of hematologic malignancy ( 113 ). The mean 25(OH)D level increased from 20.1 ng/mL to 24.3 ng/mL in the CaD group and decreased from 20.8 ng/mL to 18.2 ng/mL in the placebo group. Patients in the intervention arm had a 20% decreased risk of incident hematologic malignancies [hazard ratio (HR), 0.80; 95% confidence interval (CI), 0.65-0.99], and a 54% reduction in mortality from lymphoid malignancies (HR, 0.46; 95% CI, 0.24-0.89).

In contrast, recent RCTs failed to show a reduction in the risk of incident cancer with vitamin D supplementation ( 114 , 115 ). Lappe and cols. randomized 2,303 healthy postmenopausal women aged ≥ 55 years from 31 rural counties to receive vitamin D 2,000 IU plus calcium 1,500 mg/day or placebo. The participants were allowed to take up to 800 IU per day of vitamin D supplementation, outside the intervention. The mean 25(OH)D concentration increased from 32.8 ng/mL at baseline to 43.9 ng/mL at 1 year in the active treatment group and remained unchanged in the placebo group. Over the 4-year study period, a new diagnosis of cancer was confirmed in 3.9% of patients in the vitamin D plus calcium group compared with 5.6% in the placebo group, a nonsignificant difference (p = 0.06) ( 114 ). Similarly, a *post hoc* analysis of the ViDA study, which was originally designed to assess the effect of vitamin D supplementation on the incidence of cardiovascular disease, examined whether high-dose vitamin D supplementation was associated with a reduction in cancer incidence and cancer mortality ( 115 ). In this RCT, 5,110 community-dwelling adults (mean age 66 years) received an initial 200,000 IU bolus of oral vitamin D_3_, followed by monthly doses of 100,000 IU or placebo for a median of 3.3 years. The mean level of 25(OH)D, measured in a subgroup of participants, was 25.3 ng/mL at baseline and increased to up to 54 ng/mL in the vitamin D group, being consistently greater than 20 ng/mL than the mean level in the placebo group. There was no difference in cancer incidence or cancer mortality between vitamin D and placebo arms. In another small study, 417 adult patients with digestive tract cancers were randomized to receive vitamin D (2,000 IU/day) or placebo ( 116 ). Over a median follow up of 3.5 years, the percentage of cancer relapse or death was similar between the groups. In the subgroup of patients with baseline serum 25(OH)D levels between 20 ng/mL and 40 ng/mL, the 5-year relapse-free survival was greater in the vitamin D group (HR 0.46; 95% CI, 0.24-0.86). Finally, data from VITAL, a randomized placebo-controlled trial including more than 25,000 subjects (men and women older than 50 and 55 years, respectively), also demonstrated that vitamin D (2,000 IU per day) and omega-3 supplementation did not prevent cancer and cardiovascular diseases ( 117 ). Over a follow-up of 5.3 years, cancer was diagnosed in 1,617 subjects, and no difference was observed in the incidence of cancer between the vitamin D group and the placebo group. Although this large study demonstrated that supplementation with vitamin D did not reduce the incidence of invasive cancer, *post hoc* analyses excluding the first years of follow-up showed that the rate of death from cancer was significantly lower in patients receiving vitamin D than in those on placebo. It is important to point out that the majority of the patients had normal serum 25(OH)D levels at randomization (mean ± standard deviation 30.8 ± 10.0 ng/mL), which suggests that vitamin D requirement for cancer prevention was probably already met in most participants. In contrast, 25(OH)D levels were below 20 ng/mL in 12.7% of the participants and between 20 to 30 ng/mL in 32.2% of them.

In summary, while evidence from *in vitro* studies indicates that vitamin D has antineoplastic actions, clinical trials have not shown a role for vitamin D supplementation in reducing the incidence of cancer. Current guidelines have not proposed optimal serum levels of 25(OH)D or recommended the use of vitamin D supplementation to prevent cancer or reduce cancer death ( 5 , 118 ). However, cancer is a life-threatening disease, and some data support vitamin D supplementation in reducing cancer-related mortality ( 119 ). Thus, it seems reasonable to maintain optimal vitamin D levels in individuals with a recent diagnosis of cancer and in patients on adjuvant endocrine therapy leading to bone loss.

## DRUGS THAT INTERFERE WITH VITAMIN D LEVELS

Long-term exposure to glucocorticoids is associated with an increased risk of bone loss and fractures ( 120 , 121 ). The detrimental effect of glucocorticoids on bone occurs rapidly and can be explained by direct and indirect effects, including vitamin D deficiency due to increased 25(OH)D catabolism ( 122 - 124 ). The 2017 American College of Rheumatology guideline recommends a serum concentration of 25(OH)D above 20 ng/mL in patients on glucocorticoid treatment ( 124 ). However, greater 25(OH)D levels may be beneficial, and some experts endorse concentrations above 30 ng/mL ( 3 , 5 , 125 ). Despite inconclusive data, serum levels of 25(OH)D greater than 30 ng/mL are recommended, and this approach may minimize bone loss and improve the efficacy of antiosteoporotic therapy in individuals with glucocorticoid-induced osteoporosis ( 126 ).

Prolonged use of antiepileptic drugs (AEDs) increases the risk of fractures and has negative effects on mineral metabolism, which occur as early as 6 months of starting treatment, and appears to be, at least in part, mediated by vitamin D deficiency ( 127 , 128 ). Some AEDs, including phenytoin, phenobarbital, and carbamazepine, induce the cytochrome P450 (CYP3A4) system of liver enzymes, which increases the catabolism of 25(OH)D and 1,25(OH)_2_D ( 129 ). Prospective studies indicate that vitamin D supplementation may improve or maintain bone mass in AED users ( 130 , 131 ). A randomized trial has shown that the administration of vitamin D to patients on long-term AEDs increased the mean 25(OH)D level from 13.8 ng/mL to 26.3 ng/mL and improved BMD at all skeletal sites ( 131 ). Optimal serum levels of 25(OH)D for patients on AEDs have not been established, but based on these data and considering that the 25(OH)D levels measured may be 20% lower than the actual levels, concentrations close to 30 ng/mL may be beneficial.

Combination antiretroviral therapy (cART) has a negative effect on bone metabolism ( 37 , 132 ). Some drugs can lead to low BMD and fractures, including efavirenz, which is associated with a reduction in 25(OH)D levels, and tenofovir, which is related to secondary hyperparathyroidism. Efavirenz is a potent inducer of cytochrome P450 enzymes. Several of these enzymes are involved in vitamin D metabolism, and efavirenz may have the detrimental off-target effect of reducing available vitamin D substrate and active metabolites ( 133 ). Several randomized clinical trials in patients treated with cART have evaluated the effects of vitamin D supplementation on biochemical and immune markers, as well as in serum 25(OH)D levels and BMD ( 132 - 134 ). A study of 165 patients with HIV (aged 31-36 years) receiving efavirenz, tenofovir, and emtricitabine demonstrated that vitamin D supplementation increasing serum 25(OH)D levels to 55 ng/mL attenuated bone loss at the total hip observed in patients in the placebo arm, whose 25(OH)D concentration was unchanged at 25 ng/mL over the 48-week study period ( 132 ). In addition, vitamin D was associated with improved T-helper cells (Th naïve%) and decreased RNA viral load ( 134 ) and total and non-high-density lipoprotein cholesterol ( 135 ).

Other drugs that decrease 25(OH)D levels include orlistat, ketoconazole, cholestyramine, teriparatide, and PTH (1- 84 ), and patients on chronic use of these medications should have their vitamin D status assessed ( 1 ).

## HYPERVITAMINOSIS D AND INTOXICATION

Excess vitamin D increases intestinal calcium uptake, renal tubular reabsorption and bone resorption, leading to hypercalcemia and related symptoms like nausea, vomiting, weakness, anorexia, dehydration, and acute renal failure ( 136 ). Supplementation with very high doses of vitamin D may be harmful to elderly individuals and can potentially lead to falls and fractures ( 63 ).

The cutoff values for hypervitaminosis D in both adults and children are not well established in the literature. In general, 25(OH)D values are considered high when above 90-100 ng/mL, but the risk of vitamin D intoxication, characterized by the presence of hypercalcemia, is higher when the 25(OH)D values are greater than 150 ng/mL ( 1 ). Lower values, such as 75 ng/mL, have been correlated with mild hypercalcemia in children with rickets ( 137 ), suggesting that the risk of vitamin D intoxication in children may happen with lower values of 25(OH)D.

The prevalence rates of vitamin D intoxication are still very low when compared with those of vitamin D deficiency. This was shown in a study evaluating 5,527 patients, which reported rates of vitamin D intoxication and deficiency as 2.7% and 59%, respectively ( 136 ). However, several cases of vitamin D intoxication have been reported recently in the international literature, and this complication has increased by 7.8% in the last 5 years. It is important to note that most reports of vitamin D intoxication are related to the use of empirical or supraphysiological doses of cholecalciferol mainly by injection routes, as reported in a series of 16 cases in which patients used intramuscular injection of veterinary supplement containing high doses of vitamins A, D, and E ( 138 ).

The usual dose for correction of vitamin D deficiency is 50,000 IU/week. For maintenance, the dose varies from 400 to 2,000 IU/daily, depending on the age and clinical condition of the patient. Importantly, these doses are effective and safe and have not been associated with hypervitaminosis D or acute intoxication resulting in hypercalcemia ( 136 ).

## REFERENCE VALUES

Based on the above review of the literature analyzing the impact of 25(OH)D values on clinical outcomes in specific situations, the Brazilian Society of Endocrinology and Metabolism and the Brazilian Society of Clinical Pathology recommend reference values of 25(OH)D stratified according to age and individual clinical characteristics:

### 25(OH) vitamin D concentrations:

Deficiency: <20 ng/mL Adequate for the general population < 65 years: between 20-60 ng/mL Ideal*: 30-60 ng/mL Risk of intoxication: >100 ng/mL

* Recommended for individuals with vulnerable conditions: elderly and frequent fallers, post-bariatric surgery, pregnant women, individuals using drugs that interfere with vitamin D metabolism, and patients with osteoporosis, secondary hyperparathyroidism, osteomalacia, type 1 diabetes mellitus, cancer, chronic kidney disease, or malabsorption.

So far, there is no evident benefit from maintaining 25(OH)D levels above 60 ng/mL in any clinical situation (including bone and extra-skeletal outcomes). Levels of 25(OH)D > 100 ng/mL are associated with a risk of intoxication, leading to hypercalcemia and its clinical consequences.

## FINAL CONSIDERATIONS

This position statement updating the reference values of 25(OH)D calls attention to situations at risk for vitamin D deficiency and clinical conditions in which 25(OH)D levels below 30 ng/mL could have a negative impact on health. Measurement of 25(OH)D levels in all these cases is, evidently, recommended. It is worth mentioning that the recommendations by some international guidelines differ from ours ( 139 - 143 ), showing that this remains a controversial topic. The majority of these guidelines define the 25(OH)D values considered to be deficient for the general population. Our guideline offers a different approach, aimed at patients in special situations, for whom evidence shows that higher 25(OH)D concentrations may be beneficial.

Vitamin D is important for several biological functions particularly related to bone and mineral metabolism, according to solid evidence from *in vitro* , animal, and human studies. However, evidence has been reported of some effects of vitamin D on other systems (neuromuscular, immune) and cell differentiation (suggesting an association with cancer).

Randomized, double-blind, and placebo-controlled trials have been increasingly difficult to design, since due to ethical reasons, individuals with hypovitaminosis D should not receive placebo alone for a long period of time. Additionally, numerous warnings in the media about the magnitude of vitamin D deficiency and its consequences have largely reached lay and medical populations. Consequently, vitamin D supplementation has become more frequent, and it is increasingly difficult to find individuals with vitamin D deficiency to enroll in clinical studies.

- It is important to emphasize that the ideal 25(OH)D concentrations are still debatable, which runs counter to imprecise laboratory assays and genetic and clinical characteristics of the populations studied. Additionally, most studies measuring 25(OH)D levels have evaluated populations of elderly Caucasian women, and data on vitamin D status and consequences of vitamin D deficiency in men, other age groups, or other ethnicities are scarce.
- We suggest that the management of abnormal vitamin D levels should be based on the currently available literature and critical clinical reasoning. There is a strong consensus that 25(OH)D concentrations between 20 ng/mL and 40 ng/mL are reasonably safe. In view of conflicting results, we recommend the maintenance of concentrations above 30 ng/mL in populations with harmful consequences from vitamin D deficiency, as indicated in the literature. These measures should ensure that the patients receive the benefits of vitamin D sufficiency without the additional risk of overtreatment.

This document also highlights the actual risk of vitamin D intoxication when the supplementation exceeds the recommended doses for each clinical situation, which can have serious health consequences. Due to the absolute lack of evidence and potential risk of intoxication, we recommended 25(OH)D concentrations not to exceed 60 ng/mL in any clinical situation.
